# Comparative Fracture Properties of Four Fibre Reinforced High Performance Cementitious Composites

**DOI:** 10.3390/ma13112612

**Published:** 2020-06-08

**Authors:** Piotr Smarzewski

**Affiliations:** Department of Structural Engineering, Faculty of Civil Engineering and Architecture, Lublin University of Technology, 20-618 Lublin, Poland; p.smarzewski@pollub.pl; Tel.: +48-81-538-43-94

**Keywords:** high performance cementitious composites, steel fibre, polypropylene fibre, basalt fibre, glass fibre, strength, fracture energy

## Abstract

This study investigates the fracture properties of high performance cementitious composites (HPCC) with four different types of fibres and with volume fraction content 3%. The four fibres are steel hooked end (S), polypropylene crimped (PP), basalt chopped (B), and glass (G) fibres. The tests were carried out in accordance with the RILEM recommendations. In order to examine the fresh properties of HPCC the slump flow tests were performed. Twelve fibre reinforced HPCC beam specimens with notch were cast and tested using central point loading experiments. In addition, experimental tests of the compressive strength and splitting tensile strength were carried out. The test results made it possible to obtain representative fracture parameters, such as the equivalent strengths, residual strengths, and fracture energy of fibre reinforced HPCC. The S fibre specimens showed the best performance in terms of workability, compressive strength, tensile splitting strength, and fracture energy at large deflection. On the other hand, G fibre specimens exhibited the best performance in terms of flexural strength, equivalent flexural strength at higher deflection, and residual flexural strength at lower deflection. In terms of equivalent flexural strength at lower deflection and residual flexural strength at higher deflection, basalt fibre specimens performed the best. On the contrary, polypropylene fibre reinforced beam specimens revealed the highest deflection capacity.

## 1. Introduction

The high performance cementitious composite (HPCC) is a material composed of a cementitious matrix and other fine particles such as sand, silica fume, nanosilica, fly ash, as well as granulated blast furnace slag. The densely packed structure of such a composite leads to an improvement in compressive strength, but it is also characterized by high brittleness [1]. The dispersed fibre reinforcement can effectively control crack width at the microstructure level in the entire structure and reduce the composite brittleness, and at the same time, to improve the material ductility, increase its load carrying capacity and energy absorption capacity [2,3,4,5,6]. The concept of high performance fibre reinforced cement composite (HPFRCC) is a combination of performance benefits of three innovations in the concrete technology namely fibre reinforced concrete (FRC) [7], self-compacting concrete (SCC) [8] and high performance concrete (HPC) [9] into one material with unique properties that will make HPFRCC a very valuable asset in the future to be used in the construction and civil engineering sectors. The composition of HPFRCC was developed in such a way as to ensure a balance of fibre pull-out and crack toughness. For this reason, after the formation of the first crack, the fibres could guarantee the redistribution of crack stress and allow the formation of new multiple cracks while controlling the opening of the previously occurred ones, up to the unstable location of the major damage crack [10]. Thanks to this, the composite is able to disperse a single crack into a series of tightly spaced cracks with a narrow opening whose width is less harmful to the structural durability [9]. An important aspect of fibre reinforced HPCC is its ability to resist fracture due to the fibres being discontinuous and randomly dispersed within a cementitious matrix. Knowledge of fracture parameters is necessary for appropriate use of the material in structural elements [11,12,13,14]. Fracture performance depends on many factors, such as the mechanical properties of cementitious matrix and fibre, interface properties, fibre geometry, fibre volume content, fibre direction, and bond strength [1,6,15]. Discontinuous fibres of steel, polypropylene, carbon, glass, or basalt are applied to the composite, and therefore the key parameters affecting the efficiency and cost of HPCC are the type and quantity of fibres [3].

In recent years, many researchers have concentrated on determining the fracture energy and fracture toughness properties of normal strength fibre reinforced concrete (FRC), high performance fibre reinforced concrete (HPFRC), and self-compacting fibre reinforced concrete (SCFRC). Most studies were carried out to determine the fracture properties of steel fibre reinforced concrete with different matrix strength and different types of steel fibres, and there were reported that these fibres had a very pronounced effect on improving the fracture properties of concrete [2,3,16,17,18,19,20]. Soroushian and Bayasi [16] pointed out the effect of five different types of steel fibre on the overall performance FRC with 2% steel fibres volume content. It was noted that the general workability was independent of the fibre type except for crimped fibre. In addition, they reported that straight and crimped fibres showed poorer performance than hooked fibres. Chandrangsu and Naaman [17] compared the performance of twisted, spectra and PVA fibres, in the tensile response using different specimen sizes. It was found that the twisted fibres achieved the best performance in tensile tests. In addition, a strong size effect on the strength and deflection was observed, particularly in the bending test.

The effect of glass and basalt fibres addition of on the fracture energy of FRC was investigated by Arslan [21] using fibres with a length of 24 mm and contents of 0.5, 1, 2 and 3 kg/m^3^. The results showed that the effect of fibre contents on fracture energy were significant. Kızılkanat et al. [22] conducted a study on fracture energy of FRC with basalt and glass fibre and exhibited that the addition of 0.25% fibres leads to a slight increase in fracture energy, and the addition of 1% fibres results in the fracture energy improvement of about 50%. Branston et al. [23] stated that the addition of basalt fibres in various fractions had no significant effect on the post-cracking behaviour of FRC. On the other hand, Jiang et al. [24] found that basalt fibres had a greater impact on the toughness of concrete than polypropylene fibres.

Smarzewski conducted extensive research to determine the fracture parameters of basalt [6,25,26], polypropylene and steel [27,28,29] fibre reinforced HPC. It was found that, regardless of the fibre type added up to 2.5% by volume content, fibres had significant impact on the HPCs energy absorption capacity. Smarzewski also stated that steel fibre reinforced HPC better retains cracks than polypropylene fibre reinforced HPC. In contrast, basalt fibre reinforced HPC definitely the least had retain cracks.

Rheological and mechanical properties of SCFRC were investigated in many previous studies [30,31,32,33,34]. For example, Algın and Özen [31] studied the effect of basalt fibre on the fresh and hardened properties of SCC and the stability of SCC. Çelik and Bingöl [33,34] studied the impact of the type and volume content of basalt, glass and polypropylene fibres on the rheological and mechanical properties of SCC, including fracture properties and impact resistance. Results showed that the addition of basalt, glass, and polypropylene fibres increased the flexural strength, impact resistance, and fracture energy in all self-compacting concretes. Moreover, polypropylene fibre reinforced SCC exhibited higher impact resistance and fracture energy than SCC with the addition of basalt and glass fibres.

In general, as a result of previous studies, it can be stated that steel, polypropylene, basalt, and glass fibres increase the flexural strength and tensile strength of concrete, but do not have a clearly positive effect on compressive strength. Until now, many researchers have evaluated the HPCC fracture resistance characteristics, but different cementitious matrix compositions, different fibre types, different fibre content, and different specimen sizes were used in the experiments. Therefore, studies published to date often provide conflicting and limited data on the effect of these fibres on fracture properties, toughness and post-cracking behaviour. For these reasons, it is still necessary to conduct further research on the use of different types of fibres to assess fracture behaviour of HPCC, given that knowledge of fracture parameters is necessary to design of engineering structures.

The main objective of these studies is to determine the effect of four different types of fibres on the HPCC flexural response. Tests and analysis of results were carried out in accordance with the RILEM TC 162-TDF recommendations [35,36,37]. The investigations concern composites showing deflection-hardening behaviour at a high fibre volume content of 3%. Test results demonstrate that calculations of the flexural strength parameters of HPCC reinforced with fibres over 40 mm long and high fibre content can be extended to a higher deflection points.

## 2. Experimental Program

### 2.1. Materials, Mixture Proportions and Preparation of Specimens

The HPCC matrix used for all specimens had an average compressive strength of 82 MPa, splitting tensile strength of 5 MPa, and fracture energy of 0.1 N/mm. The impact of fibres on the HPCC properties was studied on cementitious composite with a water-to-binder materials ratio (w/csf) of 0.3. Table 1 shows the mixture proportions of all the HPCC. Portland type I cement—CEM I 52.5R (CEMEX, Chełm, Poland) (specific surface area of 484 m^2^/kg), dense silica fume (STANCHEM, Niemce, Poland) (specific surface area of 17,000 m^2^/kg), sand with a grain size of 0.125/1 mm (specific weight of 1710 kg/m^3^ and absorption coefficient of 1.9%), polycarboxylate ethers-based superplasticizer (CEMEX Admixtures GmbH, Salzkotten, Germany) (specific weight of 1070 kg/m^3^), and tap water were used in all mixtures. Fine aggregate in saturated surface-dry condition was utilized. The dosage of superplasticizer was adjusted to provide a target flow of 850 ± 10 mm for HPCC-S3%, corresponding to a SF3 flow class.

Yoo et al. [19] examined the effect of 1%, 2%, 3%, and 4% micro steel fibres contents on the material and interfacial bond properties of ultra-high performance fibre reinforced concrete. They revealed that 3% steel fibres by volume provides the best performance in terms of compressive strength, modulus of elasticity, shrinkage behaviour and interfacial bond strength. In addition, previous studies [14,25,26,27,28,29,38] showed that properties of high performance concrete and its fracture toughness parameters significantly improved with the increase of fibre volume content to 2.5%. Therefore, in this work, it was decided to investigate the HPCCs with 3% volume fraction. 

**Figure 1:**
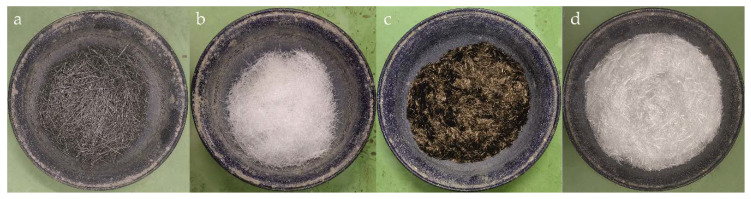
(**a**) Steel fibres, (**b**) polypropylene fibres, (**c**) basalt fibres and (**d**) glass fibres used in this study.

Four fibre types were added to the base HPCC (Table 2 and Figure 1): long steel fibres with a hooked-end shape (length of 50 mm), crimped macro-synthetic polypropylene fibres (40 mm long), short chopped basalt fibres (12 mm long), and short chopped glass fibres (18 mm long).

In this study, all the fibres exhibited varying shape and stiffness. Long steel and polypropylene fibres had a similar aspect ratio of approximately 50. On the other hand, short basalt and glass fibres had a similar diameter, elastic modulus, and tensile strength. In order to better determine the effect of fibre type, it should be noted that, aside from fibre type, all the other mixture ingredients were kept constant.

All mixtures were produced by using a laboratory rotary concrete mixer (PROMETAL, Belgrade, Serbia). The mixing sequence consisted of the following stages: dry-mixing the sand, cement and silica fume for 4 min; addition of liquid (water and superplasticizer) in the mixer and blending for next 4 min; addition of fibres and mixing for further 5 min to provide a better distribution of the fibres inside the HPCC matrix. When the mixtures were ready for casting, the consistency and flowability were determined using slump test and slump flow test, respectively. HPCC from the same batch was used to cast all the cubes and beams for each type of fibres. Thereby, 100 × 100 × 100 mm cubes and 750 × 140 × 80 mm beams were produced. Six cubes and three beams were cast for each mixture. Cubes were shaken at a rate of 150 Hz on a vibrating table as well as beams were cast from the mid-span and compacted by external vibration. In addition, the same curing procedures were adopted for all specimens. The curing regime consisted in curing the specimens covered with foil at room temperature 20 ± 2 °C for the first 48 h. After demolding, the specimens were cured in a water bath at 20 ± 2 °C until 5 days prior to testing at 28 curing days.

### 2.2. Test Set-up

Concrete workability was measured by both the slump and slump flow tests. The slump was determined according to PN-EN 12350-2 [39]. The slump flow test was carried out in accordance with the PN-EN 12350-5 standard [40] by filling the slump cone and measuring the maximum uninterrupted flow diameter in two orthogonal directions. The time for HPCC to flow 500 mm diameter circular spread (T500) was recorded. HPCC fresh properties were measured three times for each mixture as well as the values reported in Section 3.1 are the average of three measurements.

The hardened properties were evaluated after 28 days of curing. Compression and tensile splitting tests were carried out based on 100 × 100 × 100 mm cubes according to PN-EN 12390-3 [41] and PN-EN 12390-6 [42] using a load-controlled universal testing machine (CONTROLS, Milan, Italy) of 3 MN capacity. The average strengths of 3 cubes was reported for each mixture.

**Figure 2:**
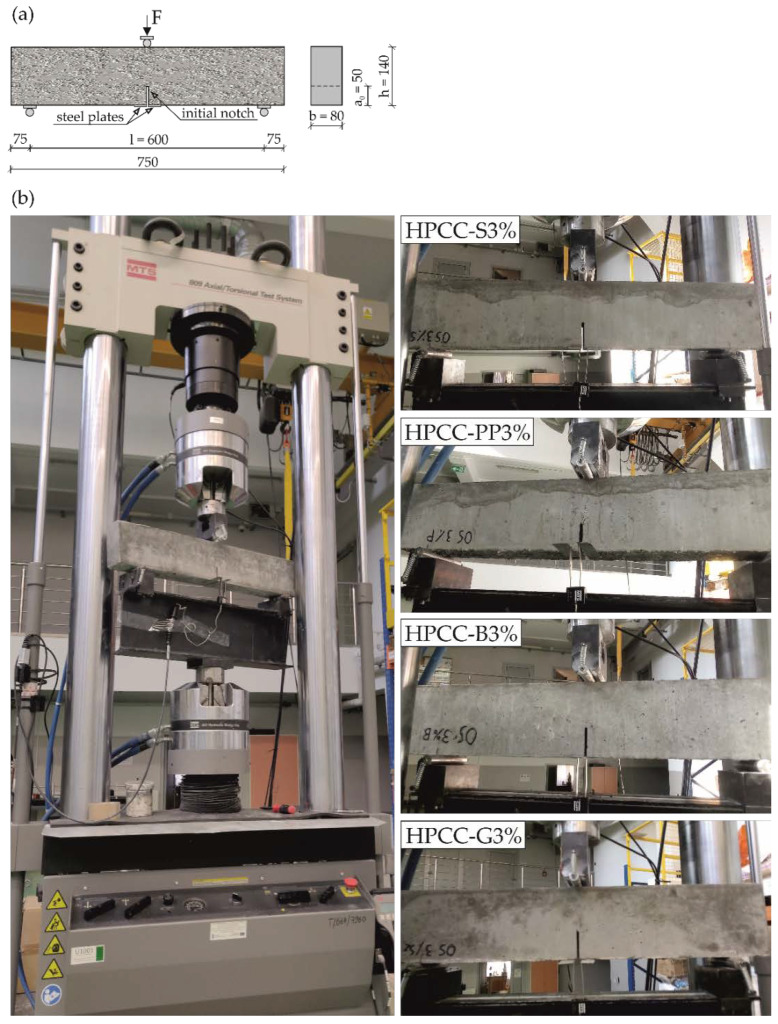
(**a**) Test specimen and (**b**) experimental set-up.

Three-point bending tests were performed in accordance with RILEM recommendations [35,36,37] on 750 × 140 × 80 mm beam specimens with a notch. An initial notch of 50 mm was made in the beam mid-span. Then the rigid steel sheets were glued at both edges of the notch. The beam specimens were carried out by a servo-hydraulic testing machine (MTS, Eden Prairie, MN, USA) in order to measure the post-cracking performance of HPCC. The load was applied continuously and monotonically. Tests were performed in a displacement controlled manner with a rate of 0.05 mm/min. The clip-gagger fixed between steel plates was used to record the crack mouth opening displacement (CMOD). The average flexural strength of 3 beams was calculated for each mixture based on PN-EN 12390-5 [43]. The geometry and dimensions of the test specimen and the experimental set-up for each type of fibre reinforced HPCC are shown in Figure 2. 

## 3. Results and Discussion

### 3.1. Influence of Fibre Type on HPCC Workability

In this study, the slump flow test was carried out to assess the flowability of HPCC. Table 3 shows the average fresh state properties, standard deviations (SD), and coefficients of variation (CV) of all tested mixtures.

**Figure 3:**
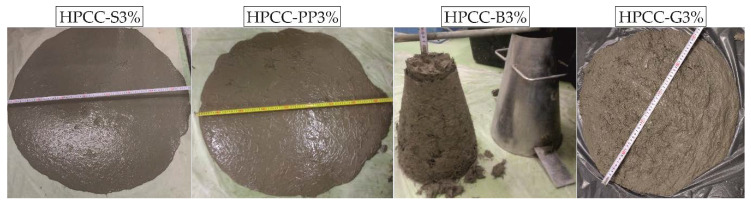
Typical slump flow/slump of HPCCs.

The HPCC mixtures design with steel, polypropylene and glass fibre achieved the slump flow spread with the value of 850, 830, and 500 mm, respectively. The slump flow values were within the range of 550–850 mm ± 50 mm which complied with the requirement of the PN-EN 12350-5 standard [40]. According to this standard, HPCC mixtures design with steel fibre and polypropylene fibre were classified as class SF3 as the slump flow results fell within the range of 760–850 mm ± 50 mm. On the other hand, HPCC mixture design with glass fibre was classified as class SF1 (the slump flow range of 550–650 mm ± 50 mm). As expected, on the basis of previous investigations [6,14,25,26], a noticeable slump reduction due to basalt fibre addition was observed, indicating stiffer, less workable HPCC-B3% mix. The slump was about of 33 mm which categorized this mixture as class S1 according to the PN-EN 12350-2 standard [39]. The reason for this phenomenon is that HPCC is dominated by a lower water-to-binder ratio (w/b), and hence there was less water in the mixing process [44,45]. In addition, basalt fibres can absorb water from the cement paste, and reduce the composite fluidity. Basalt fibres also provide additional internal friction, resulting in a reduction in the fluidity of fresh HPCC. Additionally, the fluidity of HPCC is influenced by the viscous effect of a 3D-network structure composed of a large number of short basalt fibres in the cement paste matrix. Considering the CV values given in Table 3, it can be observed that the addition of basalt fibres not only reduced slump, but also increased the scatter. It can be also observed that slump flow/slump and time (T500) results are characterized by a low coefficients of variation changing between 2.9% and 9.4%. In general, this indicates good repeatability of the measurements. Figure 3 shows slump reduction as a dependency of fibre type at 3% fibre volume content.

Steel and polypropylene fibres led to almost the same consistency in the base HPCC. By comparing these fibres versus glass fibres, it is apparent in Figure 3 that glass fibres led to a greater reduction in consistency of about 30–34% as compared to steel and polypropylene fibres reinforced HPCC. It should also be noted that the short fibres (glass and basalt) were characterized by a higher reduction in consistency as compared to the long fibres (steel and polypropylene). Upon visual inspection of the flow, it is evident that the flow shape changes from circular to elliptical with PP fibre addition content. The presence of an elongated flow shape in HPCC-PP3% can be attributed to preferential flow due to PP fibre alignment in one direction. The high stiffness of fibres likely helped to maintain a more uniform HPCC flow, while the PP fibres with the lowest stiffness interlocked and resulted in preferential flow in one direction. Additionally, the reduction of the w/b ratio due to absorbance of the water by basalt fibres in HPCC-B3% mixture led to more adverse effects by the basalt fibres on workability. Within the same fibre content, increasing the fibre length resulted in the positive impact on flowability.

### 3.2. Hardened State Properties of Fibre Reinforced HPCC

#### 3.2.1. Compressive Strength, Splitting Tensile Strength and Flexural Strength

Table 4 gives the results of the mechanical properties for all mixtures at 28 days, with standard deviations and coefficients of variation. The ratios of different HPCC strengths to the HPCC-S3% strength are also displayed in this table.

**Figure 4:**
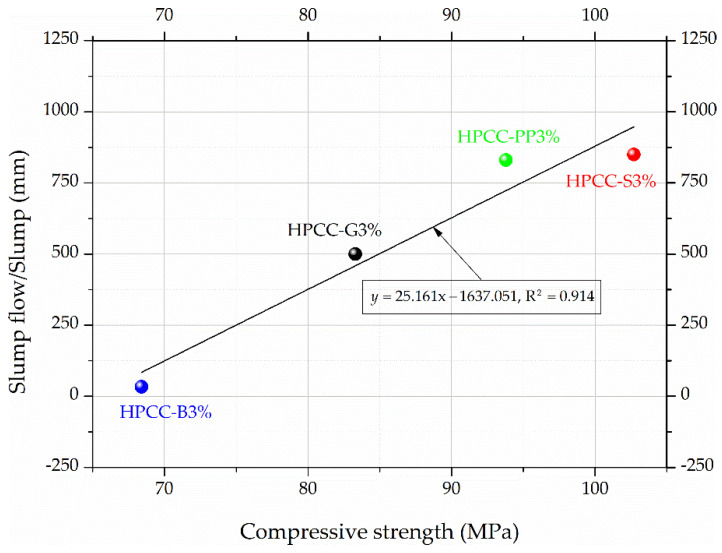
Relationship fitting curves of slump flow/slump and compressive strength of fibre reinforced HPCC.

Table 4 shows the effectiveness of fibre type on the mechanical properties of HPCC. The results indicate that the fibre type influenced the compressive strength in 3% fibre content considered. This agrees well with the results of slump, which were also affected by the incorporation of fibres type. The compressive strength of HPCC decreased approximately linearly with a decrease in the slump as shown in Figure 4. With constant fibre volume fraction of 3%, the compressive strength of HPCC was reduced by 9% (PP fibre), 19% (G fibre), and 33% (B fibre) with comparison with that of steel fibre reinforced HPCC. During the mixing procedure of the fibres and cement paste, air infiltration resulted in more pores in the cement matrix [46,47]. Also, in other studies, it was noted that, with increasing air content in mixtures with fibres, the compressive strength of concrete decreased. These results showed that, at 5.9% air content, the compressive strength was about 102 MPa, and at 8.8% air the strength dropped to about 86 MPa [48]. This means that a porous layer may form around the fibres during both mechanical compaction and free flow, which weakens the bond strength between the fibre and the HPCC matrix, which in turn may cause a decrease in compressive strength.

Jiang et al. [24] also noted a decrease in the compressive strength of normal strength concrete by 3.12% and 1.97% for specimens with polypropylene and basalt fibres, respectively, compared to reference concrete. Arslan [21] reported that the addition of 0.1% glass fibre resulted in a 1.30% reduction in the compressive strength of normal strength concrete. Algın and Özen [31] studied the effect of the addition of basalt fibres with different lengths on the properties of self-compacting concrete. The increase in basalt fibre content from 0.1% to 0.3% caused a decrease in compressive strength. Çelik and Bingöl [34] reported that SCFRC blends with a fibre content of 0.30% had lower compressive strength compared to SCFRC with a fibre content of 0.15%. The decrease in compressive strength was attributed to changes in the cement paste when basalt, polypropylene and glass fibres were added and their adverse effect on the workability of fresh SCFRC. On the other hand, Yoo et al. [19] revealed that, up to a fibre volume fraction of 3%, higher steel fibre content resulted in higher compressive strength. This was attributed to improving the fibres ability to retain the microcracks formation.

In general, HPCC tensile strength decreased as the stiffness of the fibres used decreased. An exception was noted for the glass fibre reinforced composite in which the flexural strength value was 69% higher compared to the steel fibre reinforced composite. This increase may be associated with the favorable distribution of short glass fibres in flexural samples. The hypothesis is confirmed by obtaining only 9% lower flexural strength of the composite reinforced with basalt fibres (the shortest among tested) compared to the composite with steel fibre. Concerning the tensile strength results, the splitting strength exhibited more variability and much higher results than flexural strength. A similar trend was observed in other studies different concretes and fibre types [27,38]. Banthia and Gupta [48] reported that at identical volume fractions, deformed steel macro fibres provide better strength than the crimped or self-fibrillating polypropylene macro fibres. Between the two polypropylene macro fibres, the self-fibrillating fibre performed better. The highest flexural strength was 46 MPa for the specimen with 4% fibre volume content, and this value was 44% and 14% higher than those of the specimens with fibre volume contents of 2% and 3%, respectively. Kim et al. [3] found that high strength steel twisted fibre reinforced cementitious composite showed the highest equivalent bending strength, that is, 13.1 MPa at 1.2% fibre volume content. The order of fibre performance in terms of equivalent bending strength was observed as follows: high strength steel twisted > high strength steel hooked > high molecular weight polyethylene spectra > polyvinyl alcohol (PVA). Mazaheripour et al. [30] examined the properties of hardened self-compacting lightweight concrete with polypropylene fibres and observed that the addition of 0.1%, 0.2%, and 0.3% polypropylene fibres provided an improvement in flexural strength of approximately 5%, 9%, and 11%, respectively compared to the reference specimens. Jiang et al. [15] stated a 10% improvement in flexural strength of concrete containing 0.3% basalt fibres. However, a further increase in fibre content to 0.5% has already caused a decrease in flexural strength. Çelik and Bingöl [34] noted that SCFRC with a basalt fibre content of 0.2% had the highest improvement in flexural strength by 11.6%. When the volume contents of basalt and glass fibre were increased to 0.25% and 0.3%, respectively, a slight reduction in flexural strength was observed. However, for mixtures containing polypropylene fibres, a continuous increase in flexural strength was found. 

**Figure 5:**
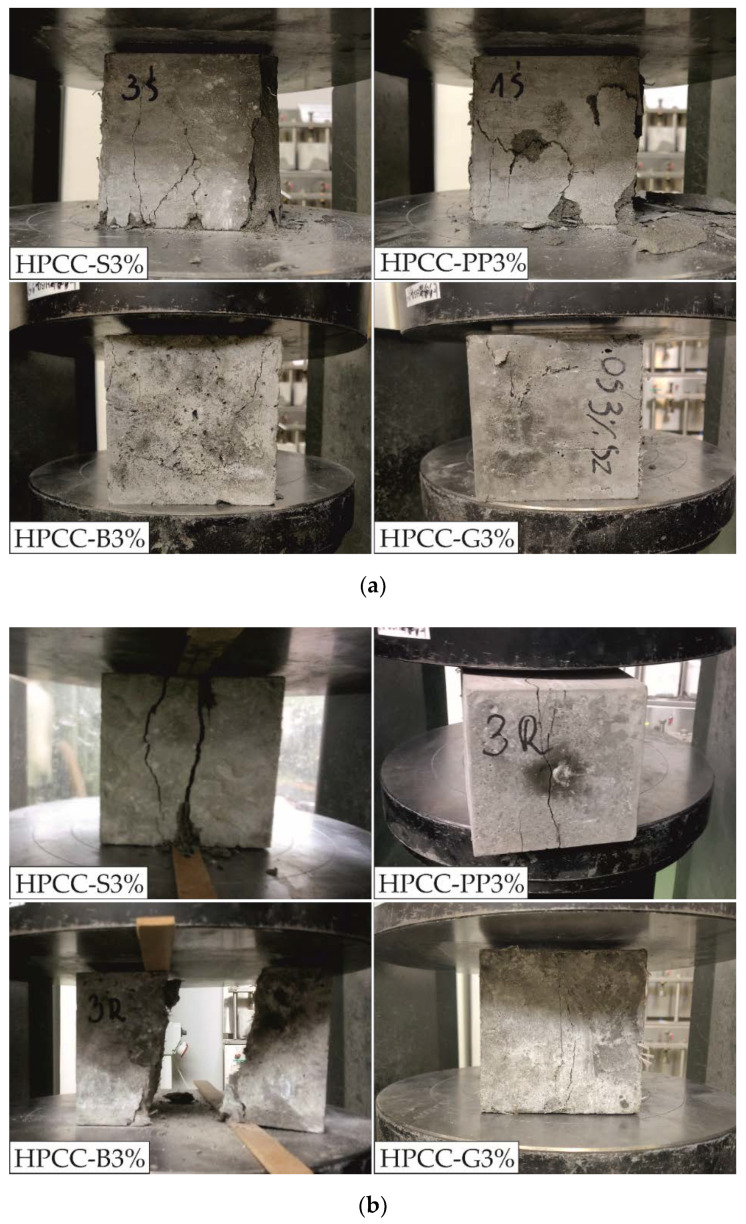
Failure modes depending on type of fibre after: (**a**) compression, (**b**) splitting tension.

Figure 5 shows failure modes of HPCC with different fibre types at 3% fibre vol. content.

The results show that the fibre type affects the failure modes of HPCC specimens both under compression and splitting tension. On the surfaces of micro fibre reinforced HPCC specimens (HPCC-B3% and HPCC-G3%), after the compression tests, small macrocracks occurred, which softened the crushing of specimens. This phenomenon is mainly attributed to the fibre bridging effect [49]. In other words, when the concrete cracks, the fibres embedded in the concrete prevent further cracking. Although micro basalt and glass fibres limited the propagation of cracks, the change in the crack direction caused the formation of other minor cracks. The incorporation of macro steel and polypropylene fibres into HPCC specimens (HPCC-S3% and HPCC-PP3%) led to the formation of wider cracks compared to micro fibre-reinforced HPCC specimens, the falling off of larger surface pieces of cementitious matrix, and dust. The failure patterns in Figure 5a show that multiple cracking occurs and some cementitious matrix pieces are extremely separated from the specimens, but still retain integrity. After compression tests, it can be concluded that micro fibre-reinforced HPCC specimens retain greater structural integrity.

As can be seen from Figure 5b, the addition of high fibre volume content (3%vol.) improved splitting strength of the HPCC matrix to varying degrees. All the specimens at 3% basalt fibre content were broken into two pieces, which accompanied by specimen debris held by fibres, small bits of debris and dust, indicating relatively brittle behaviour. For HPCC with other fibres types, the specimens were not split in pieces because the fibres bridged the two halves. It was noticed that in the specimens with steel macro fibres two major cracks were formed, in which the fibres between the two split planes can be seen. The smallest damage and narrowest cracks occurred in glass fibre reinforced specimens. This phenomenon may be caused by the stress redistribution in the HPCC matrix achieved through a dense network of glass fibres.

#### 3.2.2. Fracture Properties

**Figure 6:**
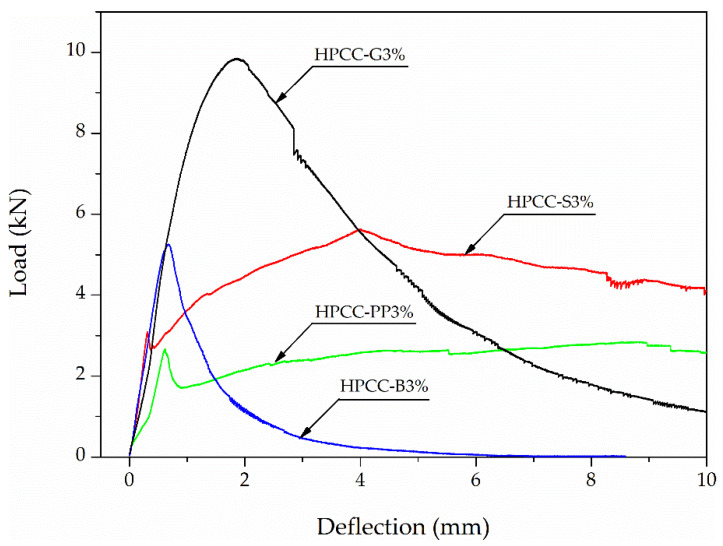
Flexural test results of HPCC reinforced four fibre types.

The flexural response of all test HPCC is shown by the load–deflection curves in Figure 6. Each curve in this figure is averaged from three specimens. Detailed average values of the parameter characterizing the flexural behaviour of HPCC with standard deviations and coefficients of variation of the test results are reported in Table 5. The ratios of HPCC flexural strength parameters and fracture energy to the HPCC-S3% strength parameters and energy are also listed in this table.

All test series with 3% fibre volume content exhibited deflection-hardening behaviour. However, different load-deflection responses were obtained. In comparing the flexural performance, the load–deflection curves show that G-fibre reinforced HPCC beam specimens produced the highest peak load compared to other HPCCs. Nevertheless, S-fibre beam specimens illustrated the highest load carrying capacity and PP-fibre beams demonstrated the best deflection capacity at peak load. The peak load for PP-fibre beams was about three and a half lower than that observed for beams with G-fibre.

**Figure 7:**
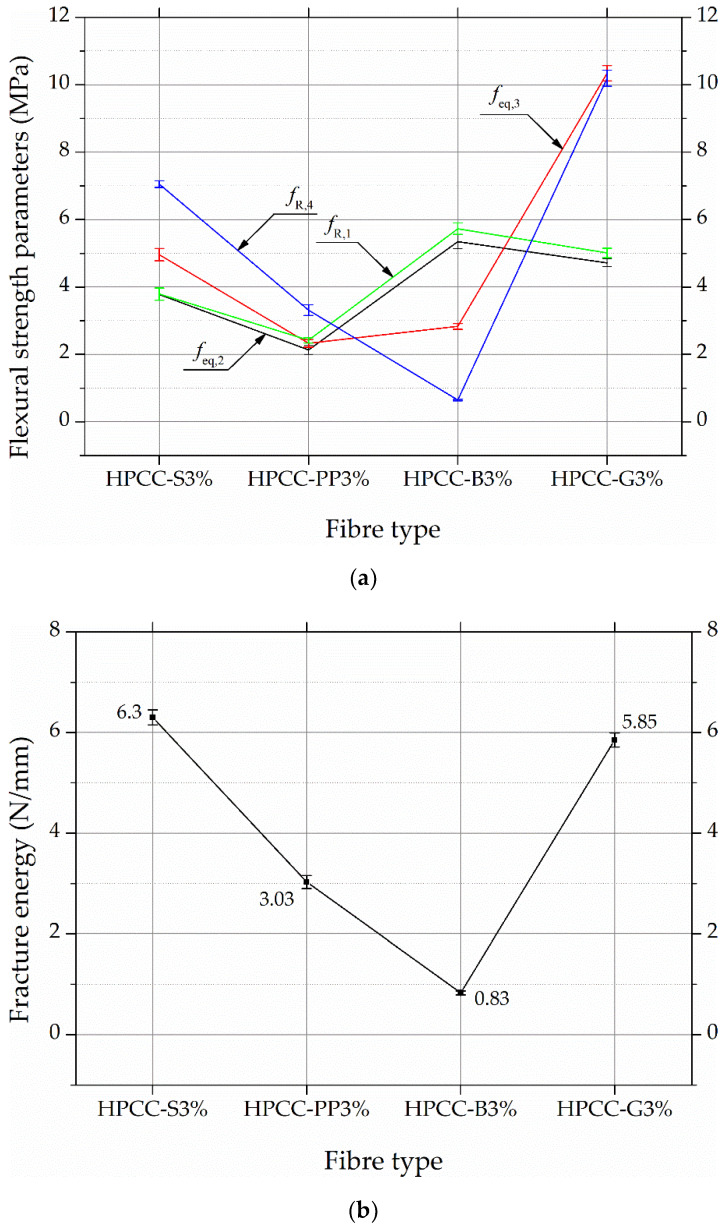
Effect of fibre type on: (**a**) equivalent and residual flexural strengths, (**b**) fracture energy.

Test results presented in Figure 6 made possible to obtain representative fracture parameters of HPCC, such as equivalent flexural tensile strengths (*f_eq,2_*, *f_eq,3_*) and residual flexural tensile strengths (*f_R,1_*, *f_R,4_*) to define amelioration after cracking for fibre reinforced concrete. Sixteen flexural tensile strength values were calculated from the load–deflection curves according to the RILEM recommendations [36,37]. Load at the limit of proportionality is equal to the load recorded up to a deflection of 0.05 mm. Equivalent strengths *f_eq,2_* and *f_eq,3_* were assessed up to a deflection of *δ_2_* = 0.7 mm and *δ_3_* = 2.7 mm, excluding energy required to fracture the plain concrete. In addition, residual strengths *f_R,1_* and *f_R,4_* were calculated at 0.46 mm and 3 mm of the deflection of beam specimen at mid-span. Fracture energy was calculated by integrating the area under the load-CMOD curve and was divided by the total crack ligament area. In accordance with [27], a reliable cut-off point can be selected at a deflection of 10 mm, and such a deflection was adopted to calculate the fracture energy in this study. The average values of flexural strength parameters and fracture energy for HPCCs are displayed in Table 5 and the effect of the fibre type on previously explained parameters is shown in Figure 7. The error indicators in the figure denote the standard deviations.

Figure 7a illustrates the development of flexural load resistance in the ascending and descending range of the load-deflection curves at 0.46, 0.7, 2.7, and 3 mm deflection points, while Figure 7b shows the influence of fibre type on the fracture energy in the test specimens with 3% fibre volume content up to the 10 mm deflection point.

For instance, the equivalent flexural tensile strength, *f_eq,2_* at 0.46 mm deflection point is 3.78 MPa for HPCC-S3%, 2.13 MPa for HPCC-PP3%, 5.35 MPa for HPCC-B3%, and 4.72 MPa for HPCC-G3%. A more obvious influence of fibre type is observed as the deflection increases up to 2.7 mm. These results show that the impact of fibre reinforcement (except for basalt fibre HPCC) is activated after the point of the limit of proportionality. Moreover, the bridging stresses are extremely dependent on the fibre type. For example, the residual flexural tensile strength, *f_R,4_*, at the 3 mm deflection point is 7.05 MPa for HPCC-S3%, 3.31 MPa for HPCC-PP3%, 0.65 MPa for HPCC-B3%, and 10.19 MPa for HPCC-G3%. The same trend is evident for the fracture energy values at the 10 mm deflection point. In terms of residual strength, *f_R,4_*, basalt fibres work the worst while glass fibres perform the best. The ratio of their strength is above fifteen. The deflection point of 3 mm was intended to specimen response in the deflection-softening range while HPCC-S3% and HPCC-PP3% beams are still in the hardening range at 3 mm. Deflection at peak load for HPCC-S3% and HPCC-PP3% is 3.98 mm and 8.83 mm, respectively, reflecting the extreme ductility of the beams. In contrast, HPCC-B3% beams show low residual strength. The breakage of basalt fibres was observed at the major crack opening. On the contrary, HPCCs with different types exhibited fibre pull-out behaviour. 

**Figure 8:**
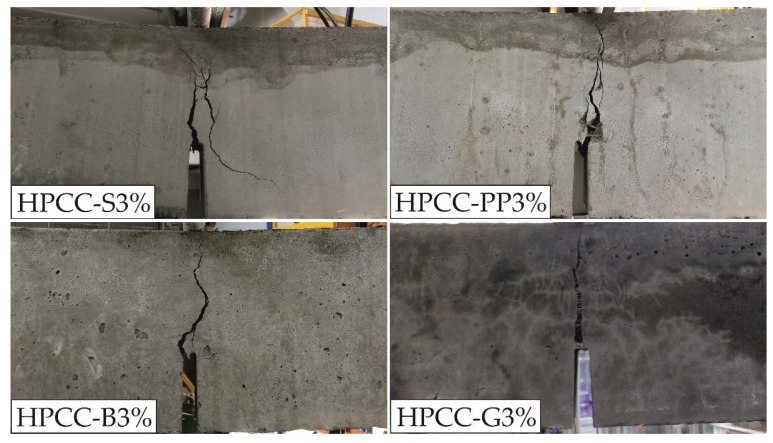
Cracking behaviour of HPCCs under bending.

Details of the cracking responses of HPCC specimens under bending are shown in Figure 8.

The cracking behaviour of HPCC specimens was investigated because it is one of the main factors describing the performance of each fibre type. It can be noticed from Figure 8 that there is quite a large variability in cracking response depending on the fibre type. All the specimens exhibited multiple cracks, except for HPCC-B3% specimens that responded in a deflection-softening manner. In general, steel and polypropylene macro fibre reinforced specimens showed more cracks than micro fibre reinforced specimens. In addition, specimens with micro fibres formed only one major crack with immediate location, although in the case of glass fibre reinforced specimens a network of multiple cracking located around the primary notch is visible. The effects of fibres bridging in the main gap are the most noticeable for specimens reinforced with glass fibres.

## 4. Conclusions

The properties of HPCC incorporating four different types of fibres (steel, polypropylene, basalt or glass) with 3% volume content in an identical matrix were herein investigated. The main findings are summarized in the following:

- The shorter the fibre, the worse the mixture workability.
- The compressive strength decreases with a slump reduction.
- The splitting and flexural tensile strength fall as the stiffness of the fibres used decrease.
- Basalt fibre reinforced beam specimens show the highest equivalent flexural strength at 0.7 mm. The order of performance in this strength level is as follows: B fibres > G fibres > S fibres > PP fibres.
- Glass fibre reinforced beam specimens generate the highest residual flexural strength at 3 mm. The order of performance in terms of residual strength is observed to be as follows: G fibres > S fibres > PP fibres > B fibres.
- Steel fibre reinforced beam specimens exhibit the highest fracture energy at large deflection of 10 mm. The order of performance at this deflection level is as follows: S fibres > G fibres > PP fibres > B fibres.
- Polypropylene fibre reinforced beam specimens demonstrate the highest deflection capacity at peak load.
- Calculations of flexural strength parameters to evaluate deflection-hardening behaviour of HPCC with 40 mm and 50 mm long fibres and with 3% fibre volume content should be extended to a larger deflection of about 10 mm.

## Figures and Tables

**Table 1:** HPCC mixture proportions.

Component	HPCC-S3%	HPCC-PP3%	HPCC-B3%	HPCC-G3%
Cement (kg/m^3^)	947.3	947.3	947.3	947.3
Silica fume (kg/m^3^)	54.2	54.2	54.2	54.2
Sand (kg/m^3^)	1042	1042	1042	1042
Superplasticizer (L/m^3^)	31.2	31.2	31.2	31.2
Water (L/m^3^)	312	312	312	312
Steel fibre (kg/m^3^)	234	—	—	—
Polypropylene fibre (kg/m^3^)	—	27	—	—
Basalt fibre (kg/m^3^)	—	—	81	—
Glass fibre (kg/m^3^)	—	—	—	75

Note: HPCC-a3%—high performance cementitious composites; a—type of fibre, where S—steel fibre, PP—polypropylene fibre, B—basalt fibre, G—glass fibre; 3%—constant fibre volume content.

**Table 2:** Properties of fibres.

Fibre Designation	S	PP	B	G
Type	Steel	Polypropylene	Basalt	Glass
Shape	Hooked-end	Crimped	Straight	Straight
Length, l (mm)	50	40	12	18
Diameter, d (mm)	1	0.75	0.013	0.014
Aspect ratio, l/d	50	53	923	1286
Density (kg/m^3^)	7800	910	2700	2500
Elastic modulus (GPa)	200	3.6	70	72
Tensile strength (MPa)	>1060	>360	>1700	>1700

**Table 3:** Fresh state properties of HPCCs.

Designation	HPCC-S3%	HPCC-PP3%	HPCC-B3%	HPCC-G3%
Slump flow/Slump (mm)	850	830	33	500
SD (mm)	35	25	2.9	20
CV (%)	4.1	3.0	8.8	2.0
T_500_ (s)	3.2	4.7	—	6.8
SD (s)	0.3	0.3	—	0.2
CV (%)	9.4	6.4	—	2.9
Class	SF3 [40]	SF3 [40]	S1 [39]	SF1 [40]

**Table 4:** Compressive, splitting tensile and flexural strengths of HPCCs.

Designation	HPCC-S3%	HPCC-PP3%	HPCC-B3%	HPCC-G3%
Compressive strength (MPa)	102.7	93.8	68.4	83.3
SD (MPa)	1.31	0.53	1.50	1.82
CV (%)	1.28	0.56	2.19	2.18
Ratio	1.00	0.91	0.67	0.81
Splitting tensile strength (MPa)	16.2	7.2	8.8	11.5
SD (MPa)	0.58	0.99	0.41	1.24
CV (%)	3.58	13.75	4.66	10.78
Ratio	1.00	0.44	0.54	0.71
Flexural strength (MPa)	7.89	3.90	7.2	13.3
SD (MPa)	0.08	0.06	0.17	0.54
CV (%)	1.01	1.54	2.36	4.06
Ratio	1.00	0.49	0.91	1.69

**Table 5:** Average values of flexural strength parameters and fracture energy of HPCCs.

Designation	HPCC-S3%	HPCC-PP3%	HPCC-B3%	HPCC-G3%
*f_eq,2_* (MPa)	3.78	2.13	5.35	4.72
SD (MPa)	0.17	0.13	0.21	0.12
CV (%)	4.50	6.10	3.92	2.54
Ratio	1.00	0.56	1.41	1.25
*f_eq,3_* (MPa)	4.96	2.33	2.83	10.34
SD (MPa)	0.18	0.11	0.09	0.23
CV (%)	3.63	4.72	3.18	2.22
Ratio	1.00	0.47	0.57	2.08
*f_R,1_* (MPa)	3.79	2.42	5.73	5.01
SD (MPa)	0.18	0.08	0.17	0.14
CV (%)	4.75	3.31	2.97	2.79
Ratio	1.00	0.64	1.51	1.32
*f_R,4_* (MPa)	7.05	3.31	0.65	10.19
SD (MPa)	0.10	0.16	0.02	0.24
CV (%)	1.42	4.83	3.08	2.36
Ratio	1.00	0.47	0.09	1.44
*G_F_* (N/mm)	6.30	3.03	0.83	5.85
SD (N/mm)	0.15	0.13	0.04	0.14
CV (%)	2.38	4.29	4.82	2.39
Ratio	1.00	0.48	0.13	0.93
